# Successful transition to mirikizumab following hepatic abscess during adalimumab therapy in a patient with ulcerative colitis

**DOI:** 10.1007/s12328-026-02397-y

**Published:** 2026-07-26

**Authors:** Kazuya Miyaguchi, Hisashi Matsumoto, Rie Shiomi, Yoshikazu Tsuzuki, Hiroyuki Imaeda

**Affiliations:** Department of Gastroenterology, Saitama Medical University, Saitama, 350-0451 Japan

**Keywords:** Ulcerative colitis, Hepatic abscess, Mirikizumab, Snti-tumor necrosis factor therapy, Biologics

## Abstract

Ulcerative colitis (UC) is associated with an increased risk of infectious complications, particularly during anti-tumor necrosis factor (anti-TNF) therapy. Pyogenic hepatic abscess is a rare but potentially life-threatening extraintestinal complication of UC. Optimal biologic management following deep infection during anti-TNF therapy remains unclear. Mirikizumab, a selective interleukin-23 p19 inhibitor, may have a more favorable infectious safety profile than anti-TNF agents. We report the case of a 19-year-old woman with pancolitis-type UC who developed persistent fever, worsening diarrhea, hematochezia, and subsequent right upper quadrant abdominal pain during maintenance therapy with adalimumab. Contrast-enhanced computed tomography revealed active colitis complicated by hepatic abscess. Adalimumab was discontinued, and intravenous ceftriaxone plus metronidazole therapy was initiated. After confirmation of infection control and abscess regression, induction therapy with mirikizumab was initiated for active UC. Clinical symptoms rapidly improved, and the patient achieved sustained clinical and endoscopic remission without recurrence of the hepatic abscess during long-term follow-up. This case suggests that mirikizumab can be successfully administered after adequate infection control in a patient with UC complicated by hepatic abscess during anti-TNF therapy. However, larger studies are needed to determine the optimal biologic option as a second-line treatment for patients requiring treatment reintroduction after deep infection.

## Introduction

Ulcerative colitis (UC) is a chronic inflammatory bowel disease characterized by relapsing intestinal inflammation, with a reported prevalence exceeding 400 cases per 100,000 persons in North America [1]. The introduction of biologic agents and small molecules has dramatically improved clinical outcomes in patients with moderate-to-severe UC [1–3]. Current therapeutic options include tumor necrosis factor (TNF) inhibitors, such as infliximab and adalimumab; α4β7 integrin inhibitors, such as vedolizumab; interleukin (IL)-12/23 inhibitors, such as ustekinumab; and Janus kinase inhibitors, such as tofacitinib [1–3]. However, infectious complications remain a major concern, particularly in patients receiving anti-TNF agents, corticosteroids, or combination immunosuppressive therapy [1–3]. A large French cohort study demonstrated that combination therapy with anti-TNF agents and immunosuppressants was associated with a significantly elevated risk of serious infections compared with anti-TNF monotherapy [3].

Hepatic abscess is an uncommon extraintestinal complication of inflammatory bowel disease (IBD) [4–9]. According to a systematic review by Alshammary et al. [4], Crohn’s disease accounted for 79.5% of reported IBD-associated hepatic abscess cases. In contrast, UC accounted for only 20.5%, indicating that hepatic abscess in UC is rarely reported. Although several cases of pyogenic hepatic abscess associated with UC have been reported, including cases caused by Staphylococcus aureus and Klebsiella pneumoniae, the condition remains exceedingly rare [10, 11].

The proposed pathogenesis involves bacterial translocation across a disrupted intestinal mucosal barrier, followed by portal venous spread to the liver during severe intestinal inflammation [5, 6, 8]. Untreated hepatic abscesses may be fatal, with a reported mortality rate of up to 8.2%; therefore, prompt diagnosis, infection control, and adequate management of intestinal inflammation are essential [4].

Mirikizumab is a humanized monoclonal antibody targeting the p19 subunit of IL-23. Selective IL-23 inhibition may have a more favorable infectious safety profile compared with anti-TNF therapy [1, 2, 12–14]. Similar to ustekinumab, mirikizumab has demonstrated relatively low rates of serious infections and favorable tolerability [1, 2, 12–14]. However, evidence regarding the use of mirikizumab after deep infection remains limited.

Here, we report a rare case of active UC complicated by a hepatic abscess during maintenance therapy with adalimumab that was successfully managed with antibiotics followed by induction therapy with mirikizumab.

## Case report

A 19-year-old woman with pancolitis-type UC was admitted to our hospital because of persistent fever, abdominal pain, watery diarrhea, and hematochezia. She had been diagnosed with UC 3 years previously and had initially achieved remission with prednisolone induction therapy, followed by maintenance treatment with 5-aminosalicylic acid (5-ASA). Two years before admission, the patient experienced a disease exacerbation requiring prednisolone at 0.5 mg/kg/day. Although a temporary improvement was achieved, the patient subsequently experienced repeated relapses and remissions. Moreover, azathioprine was discontinued because of gastrointestinal intolerance. Adalimumab therapy was initiated 1-year before admission, resulting in clinical remission. However, approximately 1 month before hospitalization, she developed persistent fever, worsening diarrhea, and hematochezia despite continued adalimumab therapy, followed by the gradual onset of right upper quadrant abdominal pain.

**Fig. 1:**
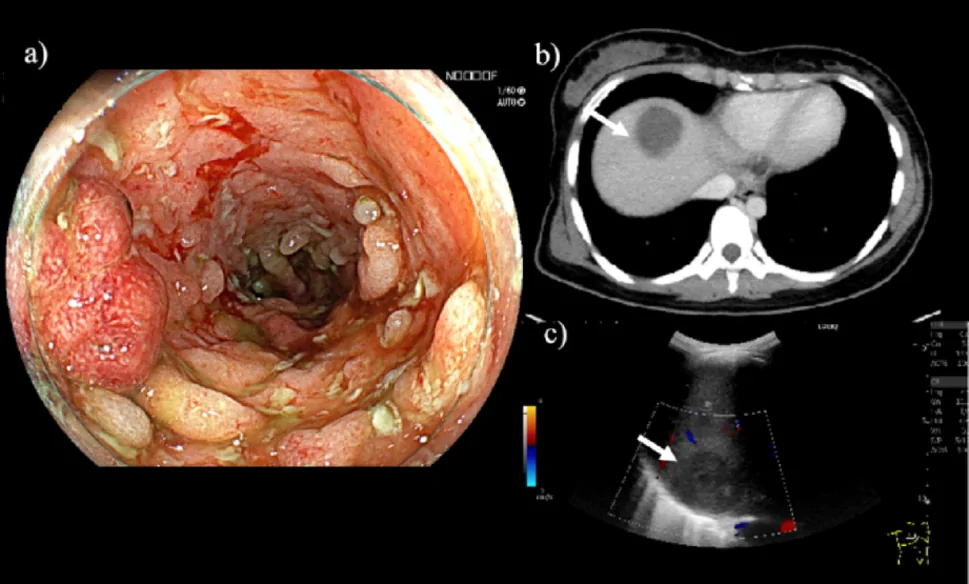
Findings at admission. **a** Colonoscopy demonstrating severe mucosal inflammation with diffuse erythema, friability, and ulceration in the sigmoid colon, consistent with a Mayo Endoscopic Subscore of 3. **b** Contrast-enhanced abdominal computed tomography demonstrating a solitary 33-mm hepatic abscess in the right hepatic lobe (arrow). **c** Abdominal ultrasound demonstrating a solitary 33-mm hepatic abscess in the right hepatic lobe (arrow)

At admission, her body temperature was 38.6 °C, pulse rate was 94 beats/min, blood pressure was 100/70 mmHg, respiratory rate was 16 breaths/min, and oxygen saturation was 98% on room air. Physical examination demonstrated diffuse abdominal tenderness without rebound tenderness. Laboratory examination demonstrated marked systemic inflammation and malnutrition, including a white blood cell count of 11,760/µL, hemoglobin level of 8.4 g/dL, platelet count of 423 × 10³/µL, C-reactive protein level of 20.15 mg/dL, albumin level of 1.9 g/dL, and erythrocyte sedimentation rate of 82 mm/h. Blood cultures were negative, and stool cultures revealed only normal intestinal flora. Colonoscopy demonstrated severe active colitis in the sigmoid colon with diffuse erythema, friability, and ulceration, consistent with a Mayo Endoscopic Subscore (MES) of 3 (Fig. 1a). The total Mayo score was 11. CMV antigenemia and CMV DNA polymerase chain reaction (PCR) testing were negative, and histological examination of colonic biopsy specimens showed no evidence of CMV inclusion bodies. The total Mayo score was 11. Contrast-enhanced abdominal computed tomography revealed diffuse colonic wall thickening and a solitary 33-mm hepatic abscess in the right hepatic lobe (Fig. 1b). No communication between the abscess cavity and the biliary or gastrointestinal tract was identified on radiological imaging. The patient had no history of sexually transmitted infections, pancreatobiliary maljunction, biliary tract disease, dental procedures, or other identifiable predisposing factors for pyogenic hepatic abscess. Immunosuppression related to adalimumab therapy, severe active colitis, and disruption of the intestinal mucosal barrier were considered the most likely contributing factors in this young patient.

**Fig. 2:**
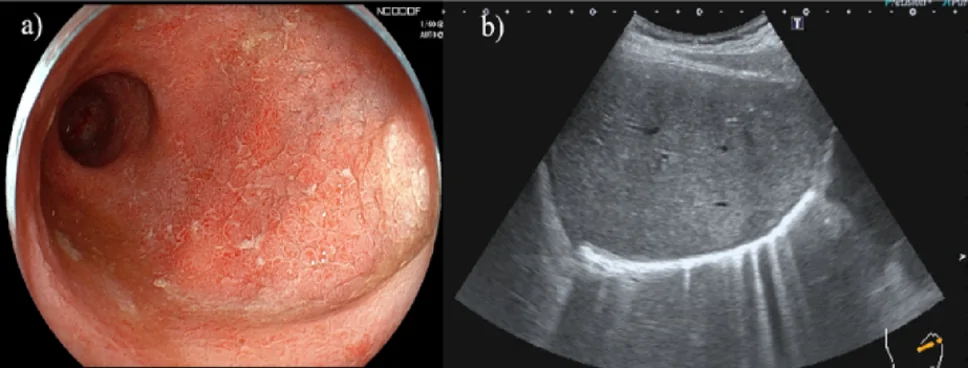
Follow-up endoscopic and imaging findings after mirikizumab therapy. **a** Colonoscopy performed 4 weeks after mirikizumab induction. **b** Abdominal ultrasonography performed 12 weeks after mirikizumab induction

Adalimumab was discontinued immediately after the diagnosis of hepatic abscess, and intravenous ceftriaxone plus metronidazole therapy was initiated. Follow-up ultrasonography performed after 2 weeks of antibiotic therapy demonstrated a reduction in abscess size. Although percutaneous transhepatic abscess drainage was considered, the lesion was located just below the diaphragm at segment 8 of the liver, making percutaneous access technically challenging; therefore, the patient opted for conservative management with antibiotics alone. Consequently, drainage was not performed. Antimicrobial therapy was continued for 3 weeks until sufficient regression of the hepatic abscess and adequate infection control were confirmed. Thereafter, mirikizumab induction therapy was initiated for active UC to achieve rapid control of persistent intestinal inflammation while minimizing the risk of recurrent systemic infection and ongoing bacterial translocation. Clinical symptoms improved within 4 weeks after mirikizumab initiation, and the patient was discharged on hospital day 25. Follow-up colonoscopy performed after 4 weeks of mirikizumab induction demonstrated marked mucosal healing with resolution of friability and ulceration, and improvement of the MES from 3 to 1 (Fig. 2a).

**Fig. 3:**
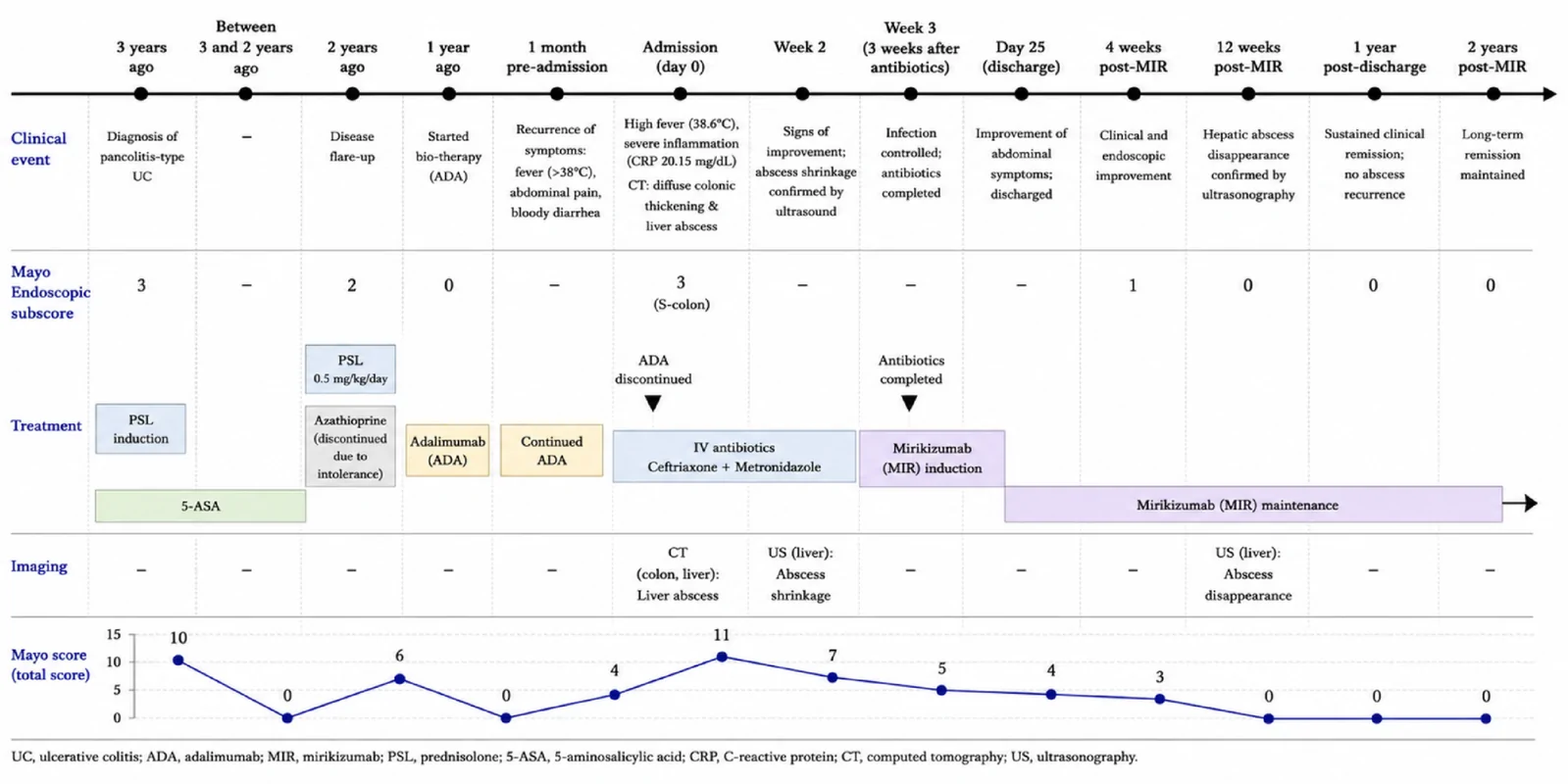
Clinical course of the patient. The patient developed active ulcerative colitis and hepatic abscess during maintenance therapy with adalimumab. After discontinuation of adalimumab and initiation of ceftriaxone plus metronidazole therapy, infection control was achieved without percutaneous drainage. Mirikizumab induction therapy was subsequently initiated, resulting in progressive improvement in Mayo score and endoscopic activity. Follow-up ultrasonography confirmed gradual regression and eventual disappearance of the hepatic abscess 12 weeks after mirikizumab initiation. Sustained clinical and endoscopic remission was maintained throughout the 2-year follow-up period

Follow-up abdominal ultrasonography performed 12 weeks after mirikizumab induction confirmed marked regression and eventual resolution of the hepatic abscess (Fig. 2b). During 2-years of follow-up, the patient maintained sustained clinical and endoscopic remission (Mayo score, 0; MES, 0) without recurrence of the hepatic abscess, while continuing mirikizumab maintenance therapy. The overall clinical course, including changes in Mayo score, endoscopic activity, treatment interventions, and hepatic abscess regression, is summarized in Fig. 3.

## Discussion

Hepatic abscess is a rare extraintestinal complication of UC [4–9]. Previous reports have suggested that hepatic abscesses in UC frequently occur during periods of active intestinal inflammation and may result from disruption of the intestinal mucosal barrier, leading to portal venous bacterial translocation and subsequent hepatic infection [5, 6, 8]. In the present case, radiological evaluation demonstrated no communication between the abscess cavity and either the biliary or gastrointestinal tract. Therefore, aggravating colonic inflammation was considered the most likely source of bacterial translocation, leading to hematogenous dissemination and subsequent hepatic abscess formation. Although anti-TNF therapy may have increased susceptibility to infection, severe active colitis and disruption of the intestinal mucosal barrier were also likely important contributors to hepatic abscess development. Blood cultures were negative in the present case. Because the abscess regressed radiologically with antimicrobial therapy, percutaneous drainage was not performed; consequently, microbiological analysis of the abscess fluid could not be obtained.

An important diagnostic consideration in this case is the distinction between pyogenic hepatic abscess and aseptic abscess syndrome (AAS), a rare neutrophilic disorder associated with inflammatory bowel disease [15, 16]. AAS is characterized by sterile abscess formation and may involve the liver, spleen, lymph nodes, or other organs. In the present case, blood cultures were negative, and microbiological confirmation could not be obtained because abscess drainage was not feasible owing to the anatomical location of the lesion. Therefore, AAS could not be completely excluded. However, several clinical findings favored a pyogenic etiology. The patient was receiving adalimumab, which may increase susceptibility to bacterial infections, and the hepatic lesion demonstrated progressive radiological regression following antibiotic therapy alone. Furthermore, no extrahepatic sterile abscesses, skin manifestations, or other clinical features suggestive of AAS were observed. Taken together, these findings support a diagnosis of pyogenic hepatic abscess, although definitive microbiological confirmation was unavailable. This diagnostic uncertainty should be considered when interpreting the clinical course and treatment response.

The clinically significant issue in this case was not only the management of hepatic abscess, but also the selection and timing of biologic reintroduction after deep infection during anti-TNF therapy. Following the diagnosis of hepatic abscess, adalimumab was discontinued, and antimicrobial therapy with ceftriaxone and metronidazole was initiated. Although interruption of immunosuppressive therapy is generally recommended in patients who develop serious infections during biologic treatment, prolonged discontinuation of therapy for UC may exacerbate intestinal inflammation, impair mucosal barrier integrity, and perpetuate infection risk through ongoing bacterial translocation [1–3]. Therefore, achieving a balance between effective infection control and timely suppression of intestinal inflammation represents a critical therapeutic challenge in such patients.

Anti-TNF agents are associated with higher rates of serious systemic infections than those observed with non-TNF-targeted biologics, particularly in patients with active disease, corticosteroid exposure, or concomitant immunosuppressive therapy [1–3]. Conversely, selective IL-23 blockade may offer a more favorable infectious safety profile [1, 12–14]. Mirikizumab selectively inhibits the p19 subunit of IL-23 and, similar to existing IL-12/23 inhibitors, has demonstrated relatively low rates of serious infection and favorable tolerability in clinical studies [1, 12–14].

In the present case, the selection of mirikizumab was based on several considerations. Although vedolizumab and ustekinumab were also considered as potential treatment options after infection control, there is currently no head-to-head evidence demonstrating the superiority of mirikizumab over these agents in this clinical setting. Nevertheless, the patient had severely active ulcerative colitis, with a total Mayo score of 11, requiring a treatment with a high likelihood of achieving clinical remission. According to the 2024 American Gastroenterological Association evidence synthesis and network meta-analysis, among biologic-experienced patients with moderate-to-severe ulcerative colitis, vedolizumab was associated with a low-certainty trivial benefit over placebo for induction of clinical remission, whereas mirikizumab demonstrated a moderate-certainty benefit over placebo. These findings supported the selection of mirikizumab in this patient with severe disease activity [17]. In addition, mirikizumab has demonstrated relatively low rates of serious infections in clinical trials [17]. Although vedolizumab remained a reasonable alternative, these considerations collectively supported the decision to initiate mirikizumab after confirmation of infection control.

Following mirikizumab initiation, the patient achieved sustained clinical and endoscopic remission, and the hepatic abscess continued to regress without recurrence. These findings suggest that effective control of intestinal inflammation with IL-23 blockade may contribute not only to UC remission but also to reduced infection recurrence associated with mucosal barrier dysfunction.

To the best of our knowledge, successful biologic reintroduction with mirikizumab after hepatic abscess complicating UC during anti-TNF therapy has rarely been reported.

This case highlights the potential clinical importance of selecting an advanced therapy with a relatively low infectious risk profile when biologic reintroduction is required after deep infection. Although this was a single case report, selective IL-23 inhibition may represent a useful therapeutic strategy for patients requiring early biologic reintroduction after adequate infection control.

In summary, we report a rare case of hepatic abscess complicating active UC during adalimumab therapy that was successfully managed with antibiotics followed by mirikizumab induction. In this patient, mirikizumab was administered without recurrent infection and achieved sustained clinical and endoscopic remission. However, conclusions regarding the comparative efficacy or safety of mirikizumab compared to other advanced therapies cannot be drawn from a single case. Further studies are needed to establish the optimal biologic strategy after deep infection in patients with UC.

Careful multidisciplinary assessment regarding infection control, abscess accessibility, disease severity, and patient preference may be important when determining the optimal timing and selection of biologic reintroduction after deep infection in UC.
